# Management of Adolescent Obesity by Pediatricians in Western Macedonia, Greece: Updated Knowledge and Daily Clinical Practice in a Geographically Challenging Region

**DOI:** 10.3390/children13070936

**Published:** 2026-07-16

**Authors:** Olga Petratou, Eleni P. Kotanidou, Anastasios Serbis, Assimina Galli-Tsinopoulou

**Affiliations:** Program of Postgraduate Studies “Adolescent Medicine and Adolescent Health Care”, School of Medicine, Faculty of Health Sciences, Aristotle University of Thessaloniki, 54124 Thessaloniki, Greece; opetrat@auth.gr (O.P.); epkotanidou@auth.gr (E.P.K.); aserbis@uoi.gr (A.S.)

**Keywords:** adolescent obesity, pediatricians, clinical practices, barriers, obesity management, multidisciplinary care

## Abstract

**Highlights:**

**What are the main findings?**

- Among pediatricians practicing in medically underserved regions, there are significant gaps in specialized training and awareness on current diagnostic criteria and evidence-based management for adolescent obesity.
- Key identified barriers in daily clinical practice include low family cooperation/adherence, lack of referral structures, difficulty engaging adolescents, and lack of guidelines or protocols reflecting both interpersonal and structural obstacles to comprehensive obesity care in this medically underserved region.

**What are the implications of the main findings?**

- There is an urgent need for continuing medical education programs for pediatricians, in adolescent obesity management, to close the identified training and knowledge gaps.
- Family engagement strategies and non-stigmatizing communication approaches must be prioritized, given that low family cooperation was the single most cited barrier, in alignment with AAP guideline recommendations to use sensitive, permission-based language and family-centered behavioral interventions to improve adherence and outcomes.

**Abstract:**

Background: Adolescent obesity is an escalating public health concern globally and in Greece, associated with significant physical and psychological consequences. Pediatricians serve as frontline clinicians in the early identification, counseling, and management of adolescents with excess weight; however, their practices, perceived competence, and the barriers they face remain insufficiently explored in certain Greek regions. Methods: A cross-sectional observational study was conducted using a structured, anonymous questionnaire distributed to all pediatricians working in Western Macedonia (*n* = 60). Fifty-one pediatricians participated (response rate 85%). The questionnaire assessed demographic characteristics, training, knowledge, clinical practices, attitudes, self-assessed competence, and perceived barriers. Results: Most participants (88.2%) reported no specialized training in adolescent obesity. Nearly half of respondents were unfamiliar with current diagnostic criteria for adolescent obesity. Although pediatricians expressed confidence in providing nutritional and lifestyle counseling, they reported limited competence in pharmacotherapy and bariatric surgery discussions. The most prominent barriers included insufficient family cooperation, lack of referral pathways, and difficulty engaging adolescents. Older and more experienced pediatricians reported higher levels of perceived competence and stronger interdisciplinary collaboration. Conclusions: Significant gaps in training and clinical support hinder the effective management of adolescent obesity in Western Macedonia. Strengthening continuous professional education, establishing multidisciplinary obesity care networks, and improving family engagement strategies appear crucial to enhancing clinical practice and improving health outcomes for adolescents.

## 1. Introduction

Childhood and adolescent overweight and obesity constitute one of the most pressing public health challenges worldwide, with steadily increasing prevalence over recent decades [1]. A recent systematic review and meta-analysis estimated that the global prevalence of overweight and obesity in children and adolescents has risen substantially since 1990, with Mediterranean countries—including Greece—consistently reporting among the highest rates in Europe [2,3]. Greece, in particular, has been identified as having one of the highest childhood and adolescent overweight and obesity prevalence rates in Europe, ranging from 36.8% to 40.8% depending on the time period and criteria applied [2]. Excess body weight before adulthood, is associated with a wide range of adverse short- and long-term health outcomes, including metabolic, cardiovascular, and psychosocial complications, as well as a significantly increased risk of obesity persisting into adulthood [4,5]. Adolescence represents a distinct developmental period, during which lifestyle habits become established, autonomy increases, and weight management-related strategies are of particular interest in order to promote well-being. Thus, early identification of body weight disorders and timely intervention to promote healthy weight are therefore critical components of effective prevention strategies for obesity throughout life.

Pediatricians play a central role in the prevention and management of childhood and adolescent overweight and obesity, particularly within primary healthcare settings. As trusted healthcare providers, they are uniquely positioned to monitor growth patterns, identify deviations from healthy weight trajectories, and counsel children, adolescents and their families on nutrition, physical activity, and lifestyle behaviors [6,7,8]. International guidelines, including the 2023 American Academy of Pediatrics (AAP) Clinical Practice Guideline and the 2024 United States Preventive Services Task Force (USPSTF) recommendation, emphasize the importance of routine anthropometrics assessment, body mass index (BMI) screening, family-centered counseling and motivational interviewing as key elements of pediatric obesity management [6,7,9]. Even more precisely, the USPSTF issued a Grade B recommendation for pediatricians to provide or refer children and adolescents aged 6 years and older with high BMI to comprehensive, intensive behavioral interventions [7]. Nevertheless, integrating these recommendations into everyday clinical practice remains challenging particularly in resource-limited or geographically underserved settings.

Previous research has shown that pediatricians’ engagement in weight management practices is influenced by multiple factors, including professional training, confidence in counseling skills, time constraints, availability of supportive resources, and perceptions regarding parental receptiveness [10,11]. A mixed-methods systematic review mapping barriers onto the Capability, Opportunity, and Motivation model of Behavior identified clinician-level factors such as lack of knowledge and skills, parent-level factors like lack of motivation, and organization-level factors such as inadequate training and time constraints as the key determinants of inconsistent implementation of recommended practices [12]. A European cross-sectional survey in primary care providers of France, Italy, Poland, and Ukraine recorded that a significant part of clinicians feels insufficiently competent to manage childhood obesity effectively, despite recognizing their critical role in obesity management [13]. A nationwide German survey among primary care pediatricians reported that nearly 41% disengaged from obesity prevention due to perceived inefficacy and lack of financial reimbursement was endorsed by over 90% of the participating pediatricians [14]. In addition, broader system-level barriers—such as limited access to multidisciplinary services and lack of structured prevention programs—may further hinder effective intervention [10,15]. As a result, substantial variability exists in how pediatricians approach childhood overweight and obesity in clinical practice.

Understanding pediatricians’ current practices, attitudes, and perceived barriers is essential for informing targeted interventions aimed at strengthening obesity prevention and management within primary care. While several international studies have explored these issues, evidence from specific national and regional contexts remains limited [12,13,14]. This gap is particularly relevant in Greece, where childhood obesity prevalence remains among the highest in Europe, since overweight and obesity rates of 19.2% and 12.1%, are reported among children aged 10–16 years in Western Greece [16]. Regional data concerning pediatric healthcare delivery and provider practices remain limited. A recent investigation has underscored notable regional disparities in the prevalence of childhood obesity, identifying in Western Greece as exhibiting the highest rates [17]. Variations in local healthcare infrastructure, professional training programs, geographic obstacles to specialist access, and sociocultural determinants are likely influential factors shaping pediatric clinical practice. These findings emphasize the critical necessity for context-specific data collection, particularly in under-resourced and underserved regions, to inform targeted interventions and healthcare policy.

The present study aims to investigate pediatricians’ practices related to the prevention and management of adolescent overweight and obesity, as well as the barriers they perceive in implementing recommended interventions in clinical settings of Western Macedonia, Greece—a geographically remote and medically underserved region with limited pediatric specialists availability. By employing an approach that captures the perspectives of the entire regional pediatric workforce (census-based sampling approach), this study seeks to provide comprehensive and context-specific evidence that may inform clinical practice improvements, professional training initiatives, and health policy strategies focused on early and effective management of adolescent overweight and obesity.

## 2. Materials and Methods

### 2.1. Study Design

A cross-sectional descriptive study was conducted to explore pediatricians’ practices, attitudes, and perceived barriers related to the prevention and management of adolescent overweight and obesity, in Western Macedonia, Greece. Data were collected using a structured, self-administered questionnaire designed specifically for the purposes of the study.

The study protocol was approved by the Bioethics Committee of the School of Medicine, Aristotle University of Thessaloniki, approval number 246/2025, with approval granted on 4 July 2025.

### 2.2. Study Population and Sampling

The study population comprised the entire population of pediatricians practicing in the Western Macedonia region of Northern Greece. Western Macedonia is a geographically remote, mountainous region with a population of approximately 260,000 inhabitants, characterized by limited healthcare infrastructure, sparse availability of pediatric specialists, and significant geographic barriers to healthcare access. These features classify the region as a medically underserved area within the Greek healthcare eco-system.

A total-population sampling approach (census-based) was employed, whereby all registered pediatricians in the region were invited to participate. According to the Western Macedonia Medical Association records, 60 pediatricians were registered and actively practicing in the region on July 2025. The study was conducted during the period of July–August 2026. Of these, 51 agreed to participate, yielding a response rate of 85% (51/60). The selected census-based design of the study, capturing the near-totality of the regional pediatric workforce, constitutes a key methodological strength of the study, as it reduces sampling error within the target population and provides a comprehensive representation of pediatric practice patterns across the entire region, rather than relying on a convenience or probability sample from a larger population. Such an approach is particularly valuable in small, well-defined populations where traditional sampling methods may introduce greater uncertainty. The specific census-based design has been previously recognized as a valid and informative methodology in healthcare research involving geographically defined or resource-limited settings [18,19]. Thus, eligible participants included all pediatricians actively involved in the clinical care of children and adolescents in Western Macedonia region. Participation was voluntary, and informed consent was obtained from all respondents prior to completion of the questionnaire.

### 2.3. Data Collection Instrument

Data were collected using a structured anonymous, self-administered questionnaire specifically developed for the purposes of this study. The questionnaire was designed specifically for the purposes of the present regional survey after reviewing the relevant international literature and previously published survey tools addressing pediatric and adolescent obesity management among healthcare professionals, including studies conducted in both European and United States settings [13,20]. The initial item pool was adapted to the Greek healthcare context and to the specific characteristics of Western Macedonia, including the organization of primary and secondary pediatric care, geographic barriers and access to multidisciplinary referral structures.

The questionnaire comprised multiple sections assessing:

(a) Sociodemographic and professional characteristics;
(b) Routine clinical practices related to growth monitoring and weight assessment;
(c) Counseling practices regarding nutrition, physical activity, and lifestyle behaviors;
(d) Attitudes and perceived confidence in managing adolescent overweight and obesity;
(e) Perceived barriers to effective prevention and management in clinical practice.

Most items were measured using Likert-type scales, allowing respondents to indicate the frequency of specific practices or the degree of agreement with attitudinal statements. The response formats included single-choice questions, multiple-choice questions, multiple-response items, 5-point self-efficacy scales, and an optional open-text response.

The questionnaire was reviewed by the research team for relevance, clarity and content appropriateness before distribution. The tool content is provided in the Supplementary Materials.

### 2.4. Data Collection Procedure

The questionnaire was distributed electronically to eligible participants, through the local Medical Associations which forwarded the survey to their members. Respondents completed the survey anonymously, and no identifying information was collected. Data collection was carried out over a predefined study period, from July to August 2025, during which reminders were sent through the same distribution channels to maximize response rates.

### 2.5. Ethical Considerations

The study was conducted in accordance with the principles of the Declaration of Helsinki. Ethical approval was obtained from the Aristotle University of Thessaloniki ethics committee prior to study initiation. Participation was voluntary and confidentiality was ensured.

### 2.6. Statistical Analysis

All analyses were performed using the IBM SPSS Statistics, version 29.0.2.0 software for statistics. Since the questionnaire-instrument applied in the present study was designed as a multidimensional descriptive tool assessing distinct domains of knowledge, attitudes and perceived self-efficacy rather as a single psychometric scale, no total score was calculated. All answered items were analyzed individually. Descriptive statistics were used to summarize participants’ characteristics and questionnaire responses. Categorical variables were presented as absolute and relative frequencies. Associations between categorical variables were examined using the chi-square test of independence. Fischer’s exact test was applied when expected cell counts were low. For ordinal categorical variables, including Likert-type responses, the Linear-by-Linear Association test was used where appropriate. Missing data were not imputed, and analyses were conducted using available responses for each variable. No formal adjustment for multiple comparisons was applied, given the exploratory nature of the study. Multivariable analyses were not conducted because of the limited number of observations in several subgroups. Statistical significance was set at 5%.

## 3. Results

### 3.1. Participant Characteristics

**Figure 1:**
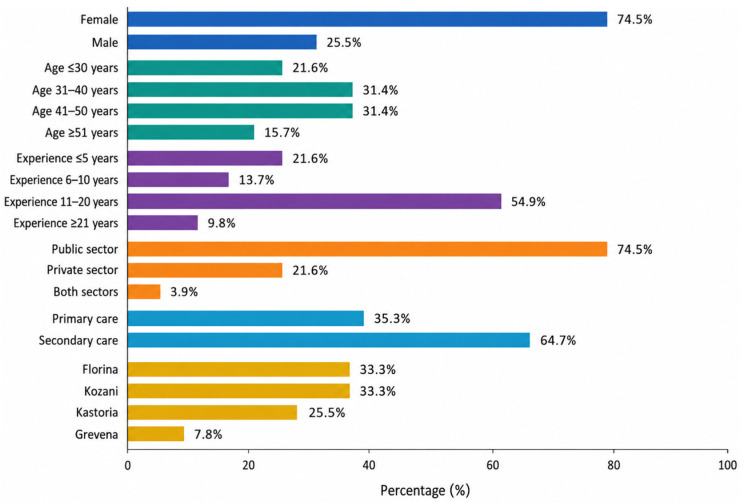
Study Sample Characteristics.

A total of 51 pediatricians practicing in Western Macedonia, Greece participated in the study, representing 85% of all registered pediatricians in Western Macedonia, Greece, effectively capturing the near-totality of the regional pediatric workforce. Most respondents were female (*n* = 38, 74.5%), while 25.5% were male (*n* = 13). Regarding age, 21.6% were ≤30 years (*n* = 11), 31.4% were 31–40 years (*n* = 16), 31.4% were 41–50 years (*n* = 16), and 15.7% were ≥51 years (*n* = 8). In terms of professional experience, 54.9% reported 11–20 years (*n* = 28), followed by ≤5 years (21.6%, *n* = 11), 6–10 years (13.7%, *n* = 7), and ≥21 years (9.8%, *n* = 5). Most participants worked in the public sector (74.5%, *n* = 38); 21.6% in the private sector (*n* = 11) and 3.9% in both (*n* = 2). Additionally, 64.7% were employed in secondary care (*n* = 33) and 35.3% in primary care (*n* = 18). Participants were distributed across the regional units of Florina (33.3%, *n* = 17), Kozani (33.3%, *n* = 17), Kastoria (25.5%, *n* = 13), and Grevena (7.8%, *n* = 4) (Figure 1).

### 3.2. Training and Knowledge Related to Adolescent Obesity

The majority of participants reported no specialized training in adolescent obesity (88.2%, *n* = 45), whereas 11.8% (*n* = 6) reported having received specialized training. Receipt of training was not significantly associated with gender (Fisher’s exact test, *p* = 0.318), age (χ^2^ = 2.231, *p* = 0.526), years of experience (χ^2^ = 2.008, *p* = 0.571), sector of employment (χ^2^ = 0.413, *p* = 0.813), healthcare level (χ^2^ = 2.931, *p* = 0.087; Fisher’s *p* = 0.168), or regional unit (χ^2^ = 1.308, *p* = 0.727).

Regarding awareness of current diagnostic criteria for adolescent obesity, 54.9% (*n* = 28) reported knowing the criteria, while 45.1% (*n* = 23) reported not knowing them (Table 1).

### 3.3. Frequency of Encountering Adolescent Obesity Cases and Assessment Practices

Most pediatricians reported encountering cases of adolescent obesity weekly or sometimes (56.9%, *n* = 29), while 25.5% (*n* = 13) reported encountering cases rarely and 17.6% (*n* = 9) reported frequent or daily exposure. In terms of assessment practices (multiple responses allowed; 113 total responses), the most frequently reported methods were BMI (*n* = 46, 40.7%) and growth curves (*n* = 42, 37.2%), followed by waist circumference (*n* = 16, 14.2%), bioimpedance or fat measurement (*n* = 6, 5.3%), and other methods (*n* = 3, 2.7%).

### 3.4. Collaboration with Other Health Professionals

More than half of the respondents reported collaborating with other health professionals in managing adolescent obesity (58.8%, *n* = 30), whereas 41.2% (*n* = 21) reported no such collaboration.

### 3.5. Attitudes Toward Adolescent Obesity

**Figure 2:**
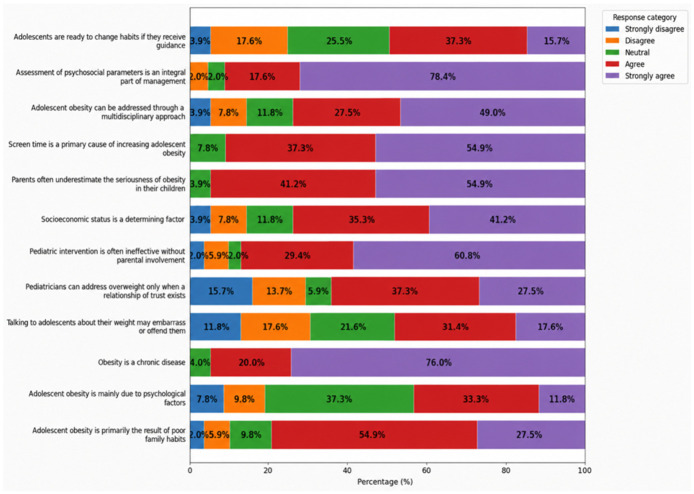
Pediatricians’ attitudes toward adolescent obesity.

Participants’ responses revealed substantial variation in pediatricians’ attitudes toward adolescent obesity. Most respondents perceived adolescent obesity primarily as a consequence of family-related lifestyle behaviors and widely acknowledged obesity as a chronic disease. At the same time, views regarding the role of psychological factors were more divided, reflecting differing interpretations of the underlying causes of obesity. Pediatricians largely recognized the importance of parental involvement and socioeconomic factors in the effectiveness of obesity management, while screen time was almost universally identified as a major contributing factor. Although many participants agreed that discussing weight issues with adolescents requires sensitivity and, in some cases, an established relationship of trust, opinions differed regarding adolescents’ readiness to change their behaviors (Figure 2).

### 3.6. Perceived Self-Efficacy in Obesity Management Practices

**Figure 3:**
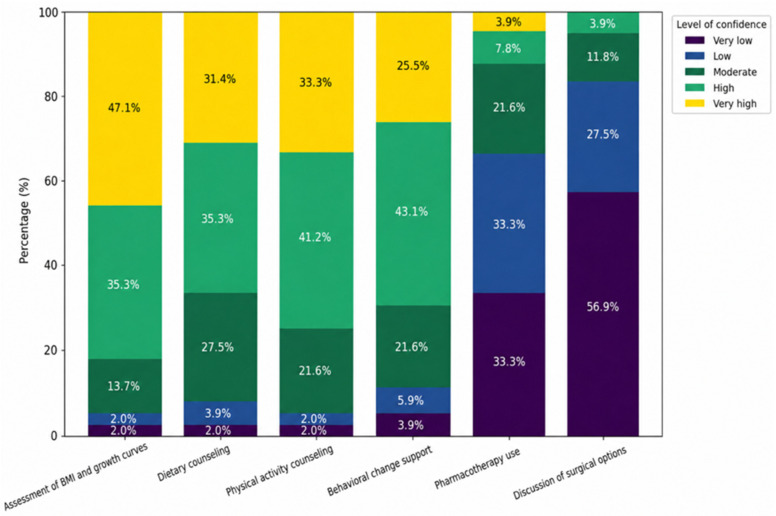
Perceived self-efficacy in obesity management practices.

Participants generally reported high perceived self-efficacy in core clinical practices related to the assessment and lifestyle management of adolescent obesity. Most pediatricians expressed strong confidence in using body mass index and growth charts for obesity assessment, as well as in providing nutritional and physical activity counseling. High levels of perceived competence were also reported with regard to motivating adolescents to adopt behavioral changes. In contrast, substantially lower confidence was observed in the use of pharmacological treatments and in discussing surgical options for obesity management, indicating a clear distinction between lifestyle-based interventions and more advanced therapeutic approaches (Figure 3).

### 3.7. Perceived Barriers in Clinical Practice

**Figure 4:**
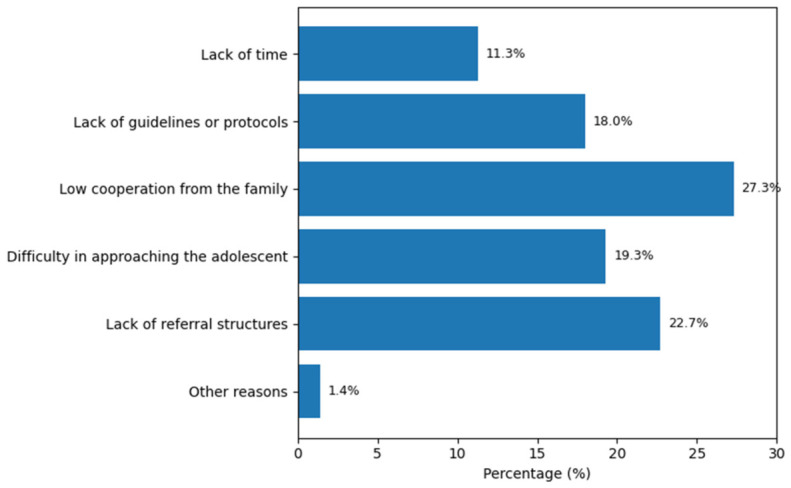
Perceived barriers in clinical practice.

Perceived barriers were reported as multiple responses (150 total responses). The most frequently endorsed barrier was low family cooperation or adherence (*n* = 41; 27.3% of responses; 80.4% of participants), followed by lack of referral structures (*n* = 34; 22.7% of responses; 66.7% of participants), difficulty approaching or engaging the adolescent (*n* = 29; 19.3% of responses; 56.9% of participants), lack of guidelines or protocols (*n* = 27; 18.0% of responses; 52.9% of participants), and lack of time (*n* = 17; 11.3% of responses; 33.3% of participants). Responses <2% were grouped as “Other reasons” (Figure 4).

### 3.8. Associations Between Demographics, Knowledge, Collaboration, and Practices

Age was positively associated with self-reported knowledge of diagnostic criteria for adolescent obesity, indicating that older pediatricians were more likely to report familiarity with current diagnostic standards (Spearman’s rho = 0.383, *p* = 0.006). Similarly, greater years of professional experience were associated with higher levels of diagnostic criteria knowledge (linear-by-linear association, *p* = 0.015). Νo significant differences were observed across regional units (χ^2^ = 1.333, *p* = 0.721).

Collaboration with other health professionals varied significantly by age group, with older pediatricians reporting higher levels of interdisciplinary collaboration compared with their younger counterparts (χ^2^ = 7.931, *p* = 0.047). The frequency with which pediatricians encountered cases of adolescent obesity did not differ across regional units (χ^2^ = 2.728, *p* = 0.842). Age-related differences were also evident in selected attitudes toward adolescent obesity. Older pediatricians were more likely to agree that obesity constitutes a chronic disease compared with younger age groups (χ^2^ = 14.292, *p* = 0.027). By contrast, perceptions regarding screen time as a major contributor to adolescent obesity did not differ significantly across age groups, indicating a broadly shared view on this factor regardless of age (χ^2^ = 12.013, *p* = 0.062).

Regarding clinical practices, significant age-group differences were identified in several behavior-related domains. Older pediatricians reported more consistent use of BMI and growth curves for obesity assessment (χ^2^ = 23.821, *p* = 0.007), greater confidence in provision of nutritional counseling (χ^2^ = 20.245, *p* = 0.010) and physical activity counseling (χ^2^ = 29.413, *p* < 0.001), and behavior change motivation (χ^2^ = 23.046, *p* = 0.033). In contrast, no significant age-group differences were observed in the perceived self-efficacy regarding the use of pharmacotherapy for obesity management (χ^2^ = 13.285, *p* = 0.384) or surgical treatment options (χ^2^ = 12.363, *p* = 0.202), suggesting uniformly low involvement in these advanced treatment strategies across all age groups.

## 4. Discussion

The present study provides insight into pediatricians’ practices, attitudes, and perceived barriers regarding the prevention and management of adolescent overweight and obesity in Western Macedonia, Greece, a geographically remote and additionally a medically underserved region. Notably, the study employed a census-based design that captured 85% of all registered pediatricians in the region, providing a comprehensive and representative picture of pediatric practice patterns rather than a sample-based estimate. The findings highlight both encouraging practices—such as routine growth monitoring and frequent nutritional counseling—and persistent challenges that limit the consistent implementation of comprehensive obesity prevention strategies in primary care. Most participating pediatricians reported regular assessment of growth parameters and widespread use of BMI to identify weight-related issues. This finding aligns with international recommendations including the 2023 AAP Clinical Practice Guideline and the 2024 USPSTF recommendation, which emphasize routine BMI screening as a cornerstone of early obesity detection [6,7,21,22].

Lifestyle counseling, especially regarding nutrition, was commonly reported, whereas counseling on physical activity and sedentary behaviors appeared less systematic. This imbalance reflects patterns observed in other healthcare settings, where dietary advice is more readily delivered than guidance on physical activity or behavior change [23,24]. A recent review on adolescent obesity emphasized that evidence-based treatment should encompass lifestyle modification, pharmacotherapy, and, when appropriate, metabolic and bariatric surgery, delivered through a comprehensive, family-centered approach [25]. Time constraints and limited consultation duration—frequently cited by respondents in this study—likely contribute to this selective counseling approach. These findings underscore the need for practical tools and brief, structured counseling strategies that can be realistically integrated into routine pediatric visits, such as the “5As” framework (ask/assess/advise/agree/assist) for pediatric obesity management, which has shown promise in improving healthcare professionals’ self-efficacy and practice behaviors [26].

Although pediatricians generally acknowledged their important role in addressing childhood overweight and obesity, confidence in managing weight issues varied considerably. Lower confidence levels, particularly among less experienced clinicians, were associated with reduced frequency of counseling practices. Previous studies have highlighted the critical role of professional training and self-efficacy in supporting effective obesity counseling, particularly through structured approaches such as motivational interviewing [20,27,28]. A recent educational intervention study demonstrated that a primary care-based obesity management curriculum significantly improved pediatric residents’ self-efficacy and documentation of obesity care [29]. This association highlights the importance of targeted professional training focused not only on knowledge acquisition but also on communication skills, motivational interviewing, and long-term behavior change strategies. The 2023 AAP CPG specifically recommends that medical schools, training programs, and professional societies improve education and training opportunities related to obesity, including training in the physiologic basis of weight dysregulation, motivational interviewing, and the social impact of obesity [6]. Strengthening pediatricians’ confidence may directly enhance the quality and consistency of obesity-related care.

Perceived barriers extended beyond individual-level factors to include broader system-level constraints. Low family cooperation and adherence was the most frequently endorsed barrier (80.4% of participants), followed by lack of referral structures (66.7%) and difficulty approaching or engaging the adolescent (56.9%). The challenge of engaging adolescents in weight-related discussions is consistent with the broader literature on weight stigma, which has documented that weight-related discussions can be uncomfortable for both clinicians and patients, and that individuals with obesity experience stigma that contributes to avoidance of healthcare services [9,11]. The AAP recommends asking permission before discussing BMI, using simple language, and employing words perceived as neutral by patients and families to facilitate non-stigmatizing conversations [6].

Notably, the structural barriers identified in this study—limited access to dietitians, psychologists, and community-based physical activity programs—reflect challenges that are not unique to Western Macedonia but have been documented across European healthcare systems. A Dutch qualitative study identified the integrated care system itself as a major barrier theme [10,15], while a German nationwide survey found that practice location and availability of local services were significant determinants of pediatricians’ cooperation behavior in obesity management [14]. However, these barriers may be amplified in Western Macedonia due to its geographic dispersion and resource constraints, making referral pathways and coordinated care less readily available than in urban centers. When intensive health behavior and lifestyle treatment programs are unavailable, the AAP recommends that pediatricians provide the most intensive program possible while building collaboration with community resources [6].

The findings of the present study are particularly relevant given the local epidemiological context of childhood obesity of the explored region. Greece has been consistently reported as having among the highest childhood overweight and obesity prevalence rates in Europe, ranging from 29 to 41% depending on the age and diagnostic criteria applied [2,3,16]. Recent data have highlighted significant regional disparities within the country [30,31,32], where Western Greece exhibits the highest overweight prevalence (24.6%) among surveyed regions [17]. The World Health Organization Childhood Obesity Surveillance Initiative data from Greece documented a decrease in obesity prevalence among boys from 30.5% in 2009–2010 to 21.7% in 2015–2017, suggesting that national-level interventions may have some effect but rates still remain high [3]. However, data from underserved areas of the country, such as Western Macedonia, remain limited and, therefore, the present study addresses this gap by providing the first comprehensive evaluation of pediatric obesity management practices in this area.

The convergence of high regional obesity prevalence, limited specialized training (88.2% of participants reported none), low awareness of current diagnostic criteria (54.9%), and significant structural barriers underscores the need for targeted interventions at both clinical and policy levels. Clinically, regionally tailored training programs incorporating motivational interviewing, the 5As framework, and structured counseling tools could enhance pediatricians’ capacity to deliver effective obesity prevention interventions [26,27,29]. From a policy perspective, strengthening primary healthcare infrastructure and investing in multidisciplinary, community-based prevention programs—as recommended by both the AAP [6] and European-level policy analyses [30]—may help overcome the systemic barriers identified in this study and promote early, sustained intervention.

Several limitations should be acknowledged. The cross-sectional design precludes causal inference, and the reliance on self-reported data may introduce reporting and social desirability bias, and also non-response bias as well. Although the questionnaire was developed based on literature and adapted to the Greek regional healthcare context, it was not formally psychometrically validated with pilot testing, expert-panel content validation, or reliability analysis of the applied questionnaire, which was based on previously published tools [13,20]. Due to cross-sectional design, the study can identify associations and describe relations but cannot establish or prove causality hypothesis. The sample size of 51 participants is numerically small, limiting the feasibility of performing multivariate analyses. Additionally, the regional focus of the study may limit generalizability to other settings.

However study sample represents 85% of all registered pediatricians in the study region, reflecting a census-based approach rather than a convenience or probability sample. This near-complete capture of the regional pediatric workforce reduces sampling error and provides a comprehensive, rather than estimated, picture of practice patterns in this defined healthcare environment. Census-based approaches have been recognized as particularly valuable in research involving small, geographically defined populations, where they offer greater validity than larger but less representative samples [18,19]. Thus, the context-specific nature of the findings represents a key strength, offering valuable insights into real-world pediatric practice within a medically underserved area—a setting that is underrepresented in the existing literature. Furthermore, the barriers and practice patterns identified in this study are consistent with those reported in larger European surveys [13,14], suggesting that the findings may have broader relevance for similar underserved regions.

## 5. Conclusions

This study highlights key aspects of pediatricians’ practices, attitudes, and perceived barriers in the prevention and management of adolescent overweight and obesity in a medically underserved region with limited pediatric specialist availability, by employing a census-based design. The findings underscore the need for targeted professional training that enhances pediatricians’ confidence and skills in obesity prevention and management, particularly in behavior change counseling. The study provides context-specific evidence from a medically underserved area that can inform clinical practice improvements and health policy initiatives aimed at promoting early, effective, and sustainable strategies for the prevention and management of adolescent overweight and obesity. Future research can explore the effectiveness of targeted training interventions and the development of care models adapted to the needs of geographically remote and resource-limited settings.

## Figures and Tables

**Table 1:** Awareness of current diagnostic criteria for adolescent obesity according to participant characteristics.

Participant Characteristic	Category	Total, *n*	Aware of Diagnostic Criteria, n (%)	Not Aware, n (%)	*p*-Value
Gender	Male	13	5 (38.5)	8 (61.5)	0.168
	Female	38	23 (60.5)	15 (39.5)	
Age group	≤30 years	11	3 (27.3)	8 (72.7)	0.044
	31–40 years	16	7 (43.8)	9 (56.2)	
	41–50 years	16	12 (75.0)	4 (25.0)	
	≥51 years	8	6 (75.0)	2 (25.0)	
Years of professional experience	≤5 years	11	3 (27.3)	8 (72.7)	0.108
	6–10 years	7	3 (42.9)	4 (57.1)	
	11–20 years	28	18 (64.3)	10 (35.7)	
	≥21 years	5	4 (80.0)	1 (20.0)	
Sector of employment	Public	38	19 (50.0)	19 (50.0)	0.049
	Private	11	9 (81.8)	2 (18.2)	
	Public and private	2	0 (0.0)	2 (100.0)	
Healthcare level	Primary care	18	14 (77.8)	4 (22.2)	0.015
	Secondary care	33	14 (42.4)	19 (57.6)	
Regional unit	Florina	17	9 (52.9)	8 (47.1)	0.721
	Kastoria	13	8 (61.5)	5 (38.5)	
	Kozani	17	9 (52.9)	8 (47.1)	
	Grevena	4	2 (50.0)	2 (50.0)	

Data is presented as n (%), *p*-value for chi-square test among participants characteristic groups.

## Data Availability

The original contributions presented in this study are included in this article, further inquiries can be directed to the corresponding author.

## Supplementary Materials

The following supporting information can be downloaded at: https://www.mdpi.com/article/10.3390/children13070936/s1, Study Questionnaire.
