# Supplementary material for: Management of Adolescent Obesity by Pediatricians in Western Macedonia, Greece: Updated Knowledge and Daily Clinical Practice in a Geographically Challenging Region
Source: Children (Basel). 2026 Jul 16;13(7):936. doi: 10.3390/children13070936 (PMC13406703; doi:10.3390/children13070936)
Supplement: Supplementary file 1 [file children-13-00936-s001.zip › children-4380833-supplementary.pdf]

Supplementary Material: Study Questionnaire

Section A: Demographic Characteristics

1.Gender: ☐ Female ☐ Male ☐ Other

2. Age: ☐ ≤30 ☐ 31-40 ☐ 41-50 ☐ ≥51

3. Years of professional experience: ☐ ≤5 ☐ 6–10 ☐ 11–20 ☐ ≥21

4. Sector of employment: ☐ Public ☐ Private ☐ Both

5. Level of healthcare employment: ☐ Primary care ☐ Secondary care

6. Regional unit of employment: ☐ Kozani ☐ Florina ☐ Kastoria ☐ Grevena

Section B: training in obesity-related topics

7. Have you received specialized training in adolescent obesity? ☐ Yes ☐ No

8. If yes, how many hours do you estimate you have devoted to such training? ☐ <5 hours ☐ 5–10 hours ☐ 11–20 hours ☐ >20 hours

Section C: knowledge and practices

9. Are you familiar with the current diagnostic criteria for adolescent obesity?

☐ Yes ☐ No

10.How often do you manage cases of adolescent obesity?

☐ Rare ☐ Sometimes/Weekly ☐ Often/Daily

11.Which assessment methods do you use? ☐ BMI ☐ Waist circumference

☐ Body fat measurment ☐ Growth charts ☐ Other

12. Do you collaborate with other healthcare professionals, such as dieticians, psychologists etc.?

☐ Yes ☐ No

Section D: perceptions and attitudes

13. Adolescent obesity is mainly the result of poor family habits.

☐ Disagree ☐ Rather disagree ☐ Neither disagree nor agree

☐ Rather agree ☐ Agree

14. Adolescent obesity is mainly due to psychological factors.

☐ Disagree ☐ Rather disagree ☐ Neither disagree nor agree

☐ Rather agree ☐ Agree

15. Obesity is a chronic disease.

☐ Disagree ☐ Rather disagree ☐ Neither disagree nor agree

☐ Rather agree ☐ Agree

16. If I talk to adolescents about their weight, I might offend or hurt them.

☐ Disagree ☐ Rather disagree ☐ Neither disagree nor agree

☐ Rather agree ☐ Agree

17.Pediatricians can discuss the issue of excess weight only when a relationship of trust has already been established.

☐ Disagree ☐ Rather disagree ☐ Neither disagree nor agree

☐ Rather agree ☐ Agree

18.Pediatricians' intervention in adolescent obesity is often ineffective without parental involvement.

☐ Disagree ☐ Rather disagree ☐ Neither disagree nor agree

☐ Rather agree ☐ Agree

19. Socio-economic status is a determining factor in adolescent obesity.

☐ Disagree ☐ Rather disagree ☐ Neither disagree nor agree

☐ Rather agree ☐ Agree

20. Parents often underestimate the severity of obesity in their children.

☐ Disagree ☐ Rather disagree ☐ Neither disagree nor agree

☐ Rather agree ☐ Agree

21. Screen time is one of the main causes of the increase in adolescent obesity.

☐ Disagree ☐ Rather disagree ☐ Neither disagree nor agree

☐ Rather agree ☐ Agree

22. Adolescent obesity can only be managed through a multidisciplinary approach.

☐ Disagree ☐ Rather disagree ☐ Neither disagree nor agree

☐ Rather agree ☐ Agree

23. Assessment of psychosocial parameters, such as self-esteem, family support, and stress, is an integral part of adolescent obesity management.

☐ Disagree ☐ Rather disagree ☐ Neither disagree nor agree

☐ Rather agree ☐ Agree

24. Adolescents are usually ready to change their habits if they receive guidance.

☐ Disagree ☐ Rather disagree ☐ Neither disagree nor agree

☐ Rather agree ☐ Agree

Section E: self-assessment

25. How confident do you feel regarding the following?

	Not at all	Slightly	Moderately	Very	Completely
Assessment of BMI and growth charts	☐	☐	☐	☐	☐
Nutritional counseling	☐	☐	☐	☐	☐
Counseling on physical activity	☐	☐	☐	☐	☐
Motivation for behavioral change	☐	☐	☐	☐	☐
Use of pharmacotherapy	☐	☐	☐	☐	☐
Discussion of surgical options	☐	☐	☐	☐	☐

Section F: barriers in clinical practice

26. What barriers do you encounter in the management of obesity? (select all that apply)

☐ Lack of time ☐ Lack of guidelines or protocols ☐ Low family cooperation ☐ Difficulty approaching the adolescent ☐ Lack of referral structures ☐ Other: ___________

Section G: personal health habits

27. Do you consider yourself to have healthy eating habits? ☐ Yes ☐ No

28. Do you exercise for at least 150 minutes per week? ☐ Yes ☐ No

29. Do you use stress management techniques? ☐ Yes ☐ No

30. Do you use mobile applications for weight monitoring? ☐ Yes ☐ No
